# Ethylene Elimination Using Activated Carbons Obtained from Baru (*Dipteryx alata* vog.) Waste and Impregnated with Copper Oxide

**DOI:** 10.3390/molecules29122717

**Published:** 2024-06-07

**Authors:** Ana Carolina de Jesus Oliveira, Camilla Alves Pereira Rodrigues, Maria Carolina de Almeida, Eliane Teixeira Mársico, Paulo Sérgio Scalize, Tatianne Ferreira de Oliveira, Victor Andrés Solar, Héctor Valdés

**Affiliations:** 1 School of Agronomy, Federal University of Goiás, Goiania 74690-900, Brazil; 2 Faculty of Nutrition, Federal University of Goiás, Goiania 74605-080, Brazil; 3 Federal Institute of Education, Science and Technology of Goiás, Inhumas 75402-556, Brazil; 4 Faculty of Veterinary, Fluminense Federal University, Niteroi 24230-231, Brazil; 5 School of Civil and Environmental Engineering, Federal University of Goiás, Goiania 74605-220, Brazil; 6 Clean Technologies Laboratory, Engineering Faculty, Universidad Católica de la Santísima Concepción, Alonso de Ribera 2850, Concepcion 4030000, Chile

**Keywords:** activated carbon, adsorption, baru waste, ethylene, porous materials

## Abstract

Ethylene is a plant hormone regulator that stimulates chlorophyll loss and promotes softening and aging, resulting in a deterioration and reduction in the post-harvest life of fruit. Commercial activated carbons have been used as ethylene scavengers during the storage and transportation of a great variety of agricultural commodities. In this work, the effect of the incorporation of copper oxide over activated carbons obtained from baru waste was assessed. Samples were characterized by X-ray diffraction (XRD), N_2_ adsorption-desorption at −196 °C, field-emission scanning electron microscopy (FESEM) coupled with energy-dispersive X-ray spectroscopy (EDS), and infrared (IR) spectroscopy. The results showed that the amount of ethylene removed using activated carbon obtained from baru waste and impregnated with copper oxide (1667 μg g^−1^) was significantly increased in comparison to the raw activated carbon (1111 μg g^−1^). In addition, carbon impregnated with copper oxide exhibited better adsorption performance at a low ethylene concentration. Activated carbons produced from baru waste are promising candidates to be used as adsorbents in the elimination of ethylene during the storage and transportation of agricultural commodities at a lower cost.

## 1. Introduction

Ethylene is a natural hormone that regulates a wide variety of developmental processes in plants and accumulates during the growth of fruit and vegetables [1]. In addition to accelerated ripening, ethylene synthesis can promote fruit softening, senescence, and rot, thereby reducing the post-harvest shelf-life of fruit with consequent economic losses [2,3]. Ethylene is physiologically active, even at low concentrations, measured in the range of parts per million (ppm) to parts per billion (ppb), which can cause the rapid deterioration of fresh agricultural commodities during transportation and storage [4].

According to the Institute for Applied Economic Research (IPEA), there is a global challenge foreseen among the twelve Sustainable Development Goals (SDGs) to halve the post-harvest losses and waste of fruit and vegetables by 2030. Thus, better management of ethylene during the storage and distribution chain becomes essential. Recent studies have reported various technologies and methods to inhibit ethylene production at the plant level or in closed fruit storage environments. Adsorption appears as one of the most promising processes to reduce ethylene content due to its low energy consumption, cost-effectiveness, and flexibility of operation [5,6]. In particular, the efficiency of the adsorption process mainly depends on the proper selection of the adsorbent [7].

To date, the main adsorbents applied for the removal of ethylene are activated carbons, zeolites, organic polymers, and organometallic structures, with an emphasis on activated carbons, which are the most used adsorption material in the elimination of Volatile Organic Compounds (VOCs) [8,9,10]. The advantages of using activated carbons (ACs) include their well-developed micropore structure, large surface area, high mechanical strength, as well as being easily regenerable materials compared to other adsorbents [10]. However, these characteristics alone may not be sufficient to ensure better efficiency in the adsorption process. One way to increase the adsorption selectivity of ACs toward target molecules is to modify their chemical surface properties accordingly [11].

The chemical modification of ACs by doping with high-valent metal species has been applied to increase adsorption selectivity toward some VOCs. First, the porous structure of ACs physically adsorbs the selected metal, and the high valence is reduced by the chemical surface groups of ACs [10]. Therefore, ACs loaded with magnesium, zinc, copper, and zirconium oxides demonstrated strong adsorption of VOCs, such as acetone and toluene, which can be explained by the acid–base interactions of VOC-metal oxide, surface functional groups, and the polarity of the adsorbate [12]. In addition, it was reported that on the surface of AC impregnated with copper oxide, some oxygen-containing functional groups were covered, which led to the generation of more surface sites, where toluene adsorption took place [13]. Another adsorption experiment demonstrated that the introduction of CuO on activated carbon can significantly improve the adsorption performance of siloxanes through hydrogen bonding [14].

So far, no work has sought to evaluate and explain the mechanism of ethylene adsorption on activated carbon doped with CuO, a common, simple, and low-cost transition metal oxide [15]. Since ACs can be prepared from different carbonaceous materials, such as agro-industrial wastes, a species of Brazilian fruit known as baru (*Dipteryx alata* vog.) is presented as a favorable source for the preparation of adsorbents. Baru is a fruit composed of a thin and rough shell (epicarp), a fleshy and fibrous pulp (mesocarp), and a single kernel that is surrounded by a woody structure (endocarp). The kernel is intended for consumption in natural form or oil extraction, while the shell and pulp, which represent around 95% of the fresh weight of the fruit, are generally discarded as waste, causing environmental impacts due to their excessive release into the environment [16,17].

In this context, this study aimed to bring a new use to wasted baru biomass, making sustainable use of baru (*Dipteryx alata* vog.) shells in the production of activated carbons impregnated with CuO species for the adsorptive removal of ethylene during the transportation and storage of agricultural commodities. The ACs were characterized physico-chemically using different techniques. Moreover, the adsorption capacities of the developed CA materials were determined through dynamic adsorption studies, and the influence of the presence of moisture during the adsorption process was evaluated. Finally, using *operando* transmission IR spectroscopy assays, an adsorption mechanism was proposed to describe the surface chemical interactions that take place between ethylene molecules and the tested ACs.

## 2. Results and Discussion

### 2.1. Characterization of the Precursor Material and the Prepared Activated Carbons

**Figure 1:**
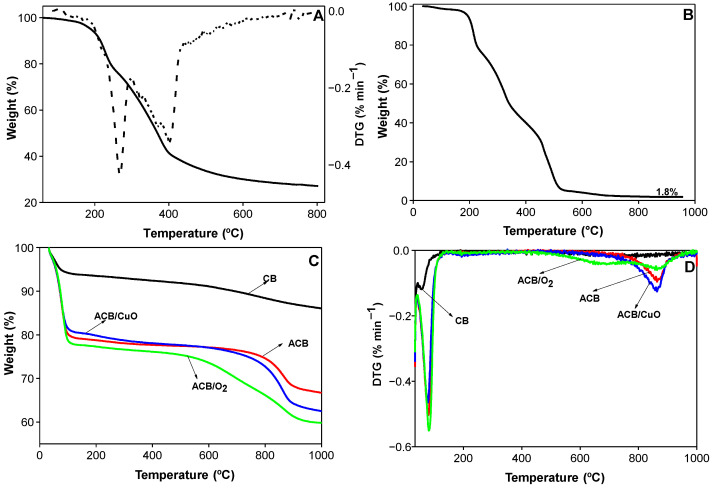
Thermogravimetric results: (**A**) solid line is the TGA curve and dashed line represents the DTG curve of baru waste, (**B**) TGA curves in air of baru waste, (**C**) TGA and (**D**) DTG curves of prepared materials: non-activated baru carbon (CB); baru activated carbon (ACB); baru activated carbon impregnated with copper oxide (ACB/CuO); and activated carbon in oxygen atmosphere (ACB/O_2_).

Figure 1A shows the thermogravimetric (TGA) curve and the first derivate of the TGA curve (DTG curve) of dried baru waste. The TGA curve displays mass loss of 73% from room temperature to 800 °C, which corresponds to a heat treatment yield of 27%.

The DTG curve in Figure 1A shows two main thermal degradation events; the first, which took place in a temperature range between 200 °C and 250 °C, can be attributed to the volatilization and/or decomposition of unsaturated triacylglycerides (oleic and linoleic acid) present in the dry baru waste. The second event, which occurred between 350 °C and 450 °C, may be related to the beginning of the decomposition of biomass, whose main components are hemicellulose, cellulose, and lignin [18,19]. The lignocellulosic fraction found in the dry baru waste was characterized by having 24% cellulose, 18% lignin, and 12% hemicellulose. Previous studies have demonstrated that precursors with high lignin content lead to the generation of ACs with higher preparation yields, whereas cellulose- rich precursors result in materials with higher surface areas [20]. In general, baru waste could be used as a precursor material for the production of porous materials due to its significant lignocellulosic content (>54%) and low ash content.

The TGA analysis in the presence of air confirms the formation of a residue (inorganic content) of around 1.8 wt.% (Figure 1B). TGA curves of prepared materials from baru waste shown in Figure 1C indicate two main stages of thermal decomposition. The first, between 40 °C and 100 °C, resulted from the desorption of water from the samples that was absorbed during the storage period. All materials, except the CB sample (non-activated baru carbon), exhibited a similar rate of thermal decomposition in the first stage. The CB sample revealed the greatest thermal stability up to the final carbonization temperature (1000 °C), with a mass loss of 13.9%, while the ACB, ACB/CuO, and ACB/O_2_ samples presented losses of 33.3%, 37.5%, and 40.2%, respectively. The thermal stability of the prepared activated carbons compared to non-activated baru carbon (CB) was significantly reduced. The main mass loss is due to the evolution of water that occurs during the condensation stage of phosphoric acid and to the reactions between phosphoric acid and the lignocellulosic fraction (hemicellulose and lignin) of the biomass, which begin around 50 °C. Since the CB material did not undergo any activation step, it was more stable to degradation, condensation, and dehydration reactions [21]. In the second stage of thermal decomposition, the ACB and ACB/CuO samples revealed similar behavior as the temperature increased from 600 °C to 900 °C. Both samples were activated with H_3_PO_4_ in an inert atmosphere; therefore, mass losses in this temperature range can be explained by carbon combustion and phosphoric acid volatilization [22]. Phosphoric acid interacts with biomass to form phosphate and polyphosphate bonds that bind and cross-link polymer fragments, decreasing the losses of volatile material during pyrolysis [23,24]. The DTG curves presented in Figure 1D confirm that the initial phase of thermal decomposition of the samples is governed by the loss of moisture, in addition to the existence of another important thermal event in the range of 750 °C to 900 °C for ACB and ACB/CuO. The maximun intensity increases in the ACB/CuO sample, indicating that the surface functional groups formed during chemical modification are less stable in this temperature range [25]. 

**Figure 2:**
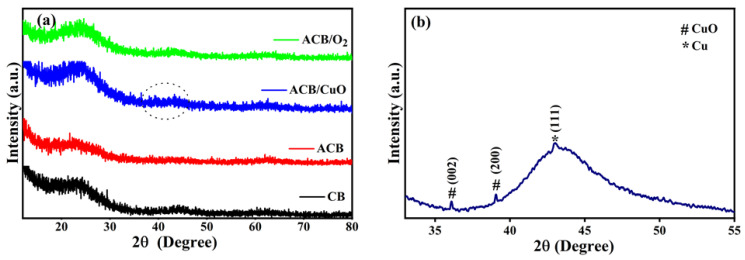
(**a**) X-ray diffraction patterns of non-activated baru carbon (CB); baru activated carbon (ACB); baru activated carbon impregnated with copper oxide (ACB/CuO); activated carbon in oxygen atmosphere (ACB/O_2_); (**b**) maximized image of the dashed circle illustrated in the X-ray diffraction pattern of baru activated carbon impregnated with copper oxide (ACB/CuO) from (**a**).

Figure 2a displays the X-ray diffraction patterns of porous materials. All AC samples show an amorphous halo at 2θ = 25°, which can be attributed to the carbon (002) plane coming from the decomposition of complex compounds present in the baru husk, such as carbohydrates, lipids, and proteins, indicating an amorphous structure [26,27].

In the diffractograms of ACB and CB, a second halo is observed at 2θ = 12.3°, indicating that the applied carbonization process at high temperature is responsible for the decomposition of some organic compounds present in the precursor matrix, consequently contributing to the formation of a semicrystalline structure. Additionally, a weak peak appeared between 35° and 45°, which corresponds to the carbon (101) plane. Despite the fact that weak copper oxide and metallic copper planes were observed in the ACB/CuO sample, due to the overlap with the carbon planes, in the 2θ range of 33°–55° (see Figure 2b), peaks related to the presence of CuO (at 36° and 39°), as well as metallic copper nanoparticles (at 42.7°), were registered. For CuO, all diffraction peaks can be indexed to the crystalline monoclinic structure of CuO nanoparticles (JCPDS card No.: 80-1916), whereas, in the case of Cu nanoparticles, they can be indexed to the face-centered cubic Cu (JCPDF Card No.: 85-1326) [28]. The carbon matrix could be responsible for hiding the intensity of such peaks [29]. Further confirmation of Cu and O elements is presented in the EDS spectra.

**Figure 3:**
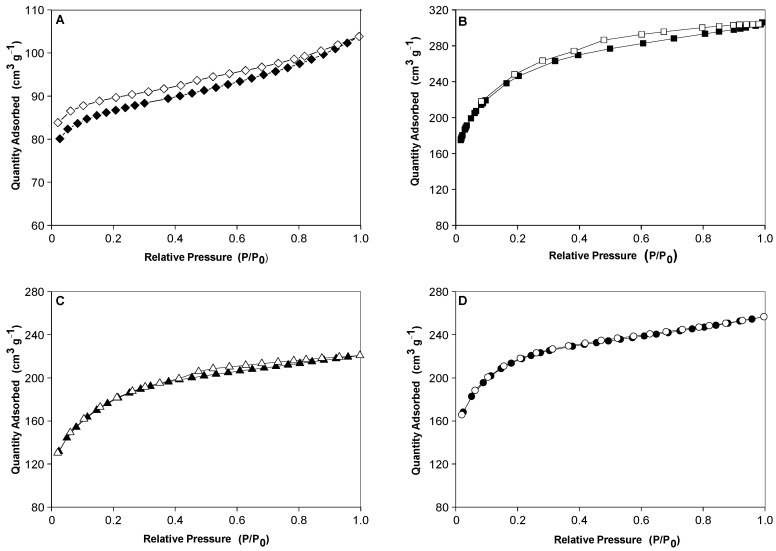
Nitrogen adsorption–desorption isotherms at −196 °C: (**A**) non-activated baru carbon (CB), (**B**) baru activated carbon (ACB), (**C**) baru activated carbon impregnated with copper oxide (ACB/CuO) and (**D**) activated carbon in oxygen atmosphere (ACB/O_2_). ♦ CB, ■ ACB, ▲ ACB/CuO, • ACB/O_2_ (filled symbols: adsorption; empty symbols: desorption).

Textural features of carbonaceous materials were analyzed by nitrogen adsorption and desorption at −196 °C, and the results are compiled in Figure 3 and Table 1. The CB material presented a type II isotherm, and the other materials (ACB, ACB/CuO and ACB/O_2_) presented type I isotherms, according to the IUPAC classification. The type I isotherm is common in adsorption measurements and occur mainly in microporous materials; the type II isotherm is characteristic of macroporous or low-pore-volume materials.

All adsorbents record BET surface (S_BET_) values between 291 and 886 m^2^ g^−1^ and a total pore volume between 0.16 and 0.46 cm^3^ g^−1^ (see Table 1). As expected, the CB sample, which did not undergo any activation step, presented a low surface area and a reduced pore volume, denoting that the chemical activation process is the main factor that contributes to the development of the microporosity and mesoporosity of the other carbons that were activated in the same proportions of precursor material and acid agent (1:2). Moreover, other factors, such as the synthesis temperature, the carbonization atmosphere (nitrogen or air), and the metal oxide used in the impregnation step, were also determining factors in the generation of the observed pore structure. The largest BET surface area (S_BET_ = 886 m^2^ g^−1^) and the greatest development of porosity and micropore volume (0.37 cm^3^ g^−1^) were obtained after chemical activation with H_3_PO_4_ (1:2) and carbonization in an N_2_ atmosphere (sample ACB). In the case of sample ACB/O_2_, which differs from the ACB sample only in terms of the carbonization atmosphere, it presented a greater volume of mesopores, which indicates that carbonization in an air atmosphere was decisive in the generation of the mesoporous structure of this material. Chatir et al. [30] indicated that atmospheric air contributes to the formation of water-soluble phosphorus compounds on the carbon surface, which allows for obtaining a porous and adjustable structure, depending on the temperature used. Loading the activated carbon surface with CuO has a significant effect on the micropore volume (0.30 cm^3^ g^−1^). Thus, compared with the activated carbon (ACB), the total pore volume, surface area, and average width of micropores decreased. It seems that the loaded CuO particles blocked part of the micropore structure, having less of an effect on the mesopores. This resulted in a decrease in the observed values of specific surface area and pore volume. The results found in the present work are comparable and even superior to other activated carbons generated from fruit wastes. For example, activated carbons produced from palm kernel/shell and activated in a nitrogen and air atmosphere showed an S_BET_ value of 457 m^2^ g^−1^ [31], while activated carbon based on lemon peel produced an S_BET_ value of around 500 m^2^ g^−1^ [32] and activated carbon derived from mangosteen peel generated S_BET_ values between 460 and 1039 m^2^ g^−1^ [33]. Regarding the applied copper impregnation process, we also observed that the textural characteristics of the ACB/CuO sample were similar and, in some cases, superior to other porous materials impregnated with copper, such as natural zeolite [5] and porous boron nitride [34], as shown in Table 2.

The surface charge distribution of carbonaceous materials was evaluated by determining the pH of the point of zero charge (pH_PZC_). The pH_PZC_ consists of the pH value at which the net charge densities on the surface of the material become zero [22]. The results for this parameter indicate that pH_PZC_ values (see Table 1) decrease with increasing precursor/acid ratios. As the activated carbon samples ACB, ACB/CuO, and ACB/O_2_ underwent the same degree of activation (1:2), very close values were found for the pH_PZC_. On the contrary, non-activated baru carbon (CB) (without any activation step) resulted in a pH_PZC_ value close to 7, indicating that at this pH value, the surface charges are neutral. Lower pH_PZC_ values denote that porous materials have a higher concentration of acidic groups, such as carboxylic and phenolic groups, resulting from the dissociation of surface oxygen complexes [35].

**Figure 4:**
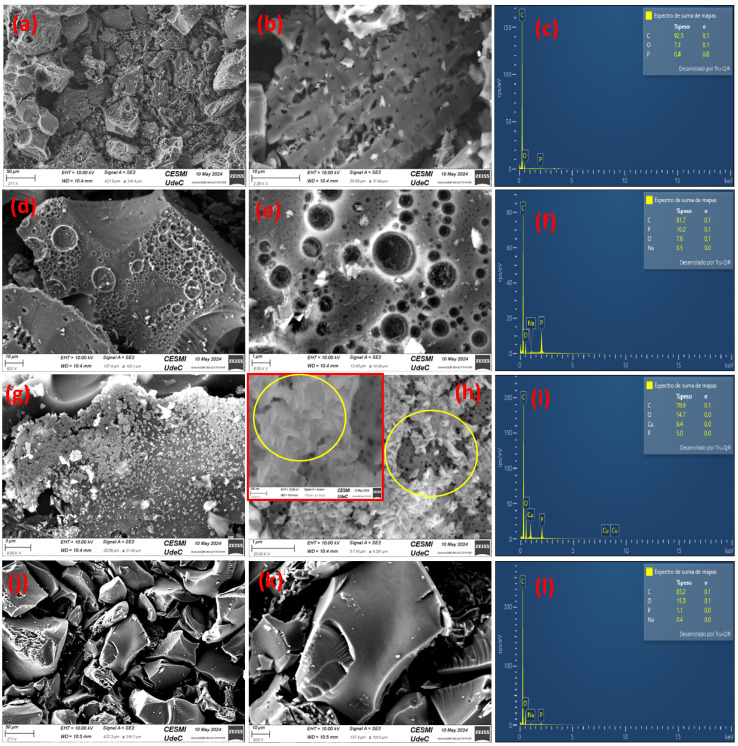
Field-emission scanning electron microscopy coupled to energy-dispersive spectroscopy of (**a**–**c**) non-activated baru carbon (CB), (**d**–**f**) baru activated carbon (ACB), (**g**–**i**) baru activated carbon impregnated with copper oxide (ACB/CuO), (**j**–**l**) activated carbon in oxygen atmosphere (ACB/O_2_).

The porous nature and elemental characteristics of the produced carbon-based materials can be clearly observed in the field-emission scanning electron microscopy (FESEM) images obtained coupled to energy-dispersive spectroscopy (EDS) (see Figure 4), which show the presence of microporous and mesoporous structures. EDS spectra indicate the presence of elements, such as carbon, oxygen, calcium, sodium, and silicon. These elements are associated with the nature of the raw material and could also be related to the applied carbonization, activation, and washing processes. Moreover, EDS analysis demonstrates the incorporation of copper on the activated carbon surface after the applied modification procedure (Figure 4i).

In Figure 4a,b, microstructural analysis of non-activated baru carbon (CB) reveals its unevenly agglomerated morphology, and the absence of porosity is confirmed. Figure 4c shows the EDS result, which confirms the presence of elemental constituents in the CB sample.

Figure 4d,e depict FESEM images of baru activated carbon (ACB), illustrating structural and porosity changes compared to the non-activated CB sample. They further confirm the successful carbon activation with good pore structures. Figure 4f displays the EDS analysis, which confirms the presence of activated carbon elements in the structures and the associated changes in elemental composition resulting from the activation process.

Figure 4g shows FESEM images of baru activated carbon impregnated with copper oxide (ACB/CuO), which exhibits uneven morphological structure and agglomerated CuO nanoparticles on the surface. A magnification image in Figure 4h further confirms the deposition of copper oxide (CuO) nanosheets on the ACB surface. An inserted image reconfirms the CuO morphology. The marked circle confirms the porosity and morphology of CuO nanosheets, showcasing the distribution of copper oxide nanoparticles over the activated carbon matrix. Figure 4i presents the EDS analysis, which reveals the elemental composition of carbon and copper within the produced material, providing information on the impregnation process and the resulting composite structure.

In Figure 4j,k, FESEM images of activated carbon in an oxygen atmosphere (ACB/O_2_) reveal the morphological changes induced by the exposure to oxygen. Figure 4l depicts the EDS analysis, which reveals the elemental composition of the activated carbon due to oxidative processes.

**Figure 5:**
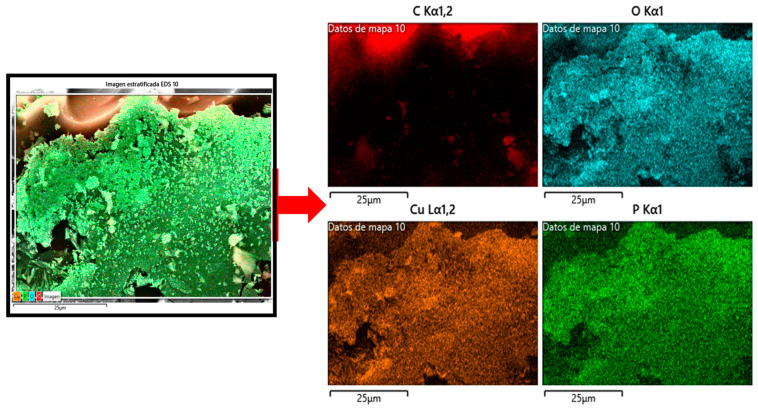
Elemental mapping analysis corresponding to the FESEM image of baru activated carbon impregnated with copper oxide (ACB/CuO).

In Figure 5, the elemental mapping analysis corresponds to the FESEM image of baru activated carbon impregnated with copper oxide (ACB/CuO). Elemental mapping analysis provides valuable information on the distribution and composition of different elements within this material. Elemental mapping reveals the spatial distribution of key elements, including carbon (C), oxygen (O), and copper (Cu), across the surface of the ACB/CuO sample. This analysis allows us to visualize the dispersion of copper oxide nanoparticles within the activated carbon matrix and evaluate the uniformity of the impregnation process. Notably, the elemental mapping highlights regions of high copper concentration, indicating the successful impregnation of copper oxide onto the activated carbon substrate.

**Figure 6:**
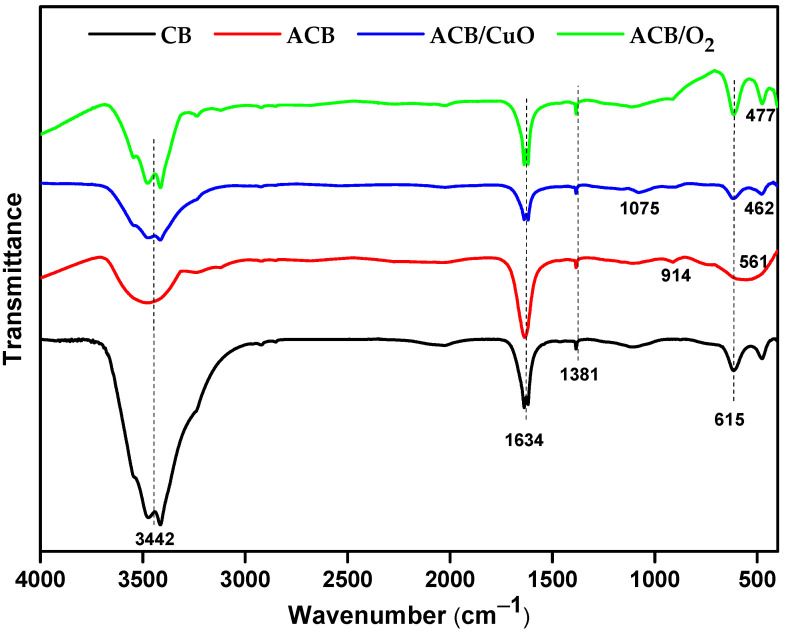
FTIR spectra of non-activated baru carbon (CB); baru activated carbon (ACB); baru activated carbon impregnated with copper oxide (ACB/CuO); and activated carbon in oxygen atmosphere (ACB/O_2_).

The surface chemistry of non-activated and activated baru carbon samples was characterized by IR spectroscopy using ATR, and the results are given in Figure 6. The characteristic IR vibrations of lignocellulosic compounds are registered at approximately 3442 cm^−1^, attributed to O-H of phenolic hydroxyl groups [36]. As can be seen from the spectra, the introduction of CuO into the ACB/CuO sample does not significantly change the surface chemistry compared to the ACB sample and to the other developed materials. The formation and shift of the IR band at 1634 cm^−1^ are due to the bending vibrations of water related to adsorbed water and remain the same for all the AC materials [5]. The most obvious differences in the vibrational spectra between ACB and ACB/CuO were evident in the interval between 400 and 700, where new IR bands attributed to stretching vibrations of the Cu-O bond of copper oxide were formed [37]. The IR bands observed in a range between 1381 cm^−1^ and 615 cm^−1^ resulted from the angular deformation of OH and angular vibration (OH) of the water molecule, respectively [38].

### 2.2. Chemical Surface Interaction Assessments by Operando IR Spectroscopy

**Figure 7:**
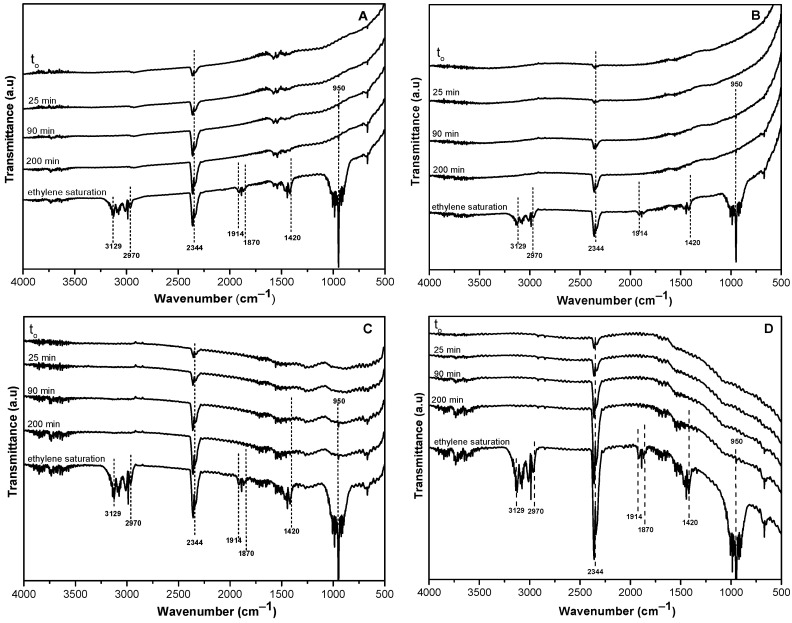
Evolution of IR spectra during ethylene adsorption on AC samples obained by *operando* transmission IR spectroscopy analyses: (**A**) non-activated baru carbon (CB), (**B**) baru activated carbon (ACB), (**C**) baru activated carbon impregnated with copper oxide (ACB/CuO), (**D**) activated carbon in oxygen atmosphere (ACB/O_2_).

Real-time analysis of the AC samples using IR spectroscopy (*operando* mode) provided useful information on the surface interactions with ethylene molecules through the adsorption process. The evolution of IR spectra is provided in Figure 7, starting at time zero and ending after 200 min of contact time, where ethylene saturation is evident in all samples. IR vibrations corresponding to ethylene gas were observed at IR bands located at 950 cm^−1^, 1420 cm^−1^, 1870 cm^−1^–1914 cm^−1^, 2344 cm^−1^, 2970 cm^−1^, and 3129 cm^−1^, which are in correspondence with the results reported by other authors [5]. It can also be noticed that the IR band observed at 3442 cm^−1^ ascribed to hydroxyl groups before coming into contact with ethylene (Figure 6) lost its intensity during continuous exposure to ethylene and was almost completed consumed. These results suggest that hydroxyl functional groups may be mainly responsible for the adsorption of ethylene molecules, changing the electrostatic potential distribution on the surface of activated carbon and facilitating important interactions with ethylene [39]. Hence, hydrogen-bonded adducts could be formed between hydroxyl functional groups on the surface AC and ethylene molecules in a similar way, as has been described for the interaction between ethylene molecules and the Brønsted acid sites of zeolitic frameworks [5]. Moreover, the mesoporous and microporous structure of all the samples allows for easy diffusion of small molecules like ethylene with a kinetic diameter of ~3.9 Å and its consequent interaction with the OH groups on the AC surface.

In the case of samples CB and ACB (Figure 7A,B), the IR absorption bands characteristic of ethylene vibrations gradually evolve as the contact time increases. In particular, the ACB/O_2_ sample (Figure 7D) exhibits more intense vibrations in the first minutes of contact time, reaching a saturation point more quickly, in which the entire surface structure of the adsorbent is covered, and well-developed bands are observed mainly between 2970–3129 cm^−1^ and 950 cm^−1^. In the case of the sample impregnated with CuO (Figure 7C), the IR band formed between 500 cm^−1^ and 700 cm^−1^ could be related to interactions of ethylene with copper incorporated on the AC surface. Experimental studies have suggested that interactions of ethylene with metal oxides could occur through the π electrons of the C=C bonds of ethylene and the metal orbitals present in the supporting structure. This is a type of interaction known as σ-donation, in which the π molecular orbital of the adsorbed ethylene donates electron density to the empty s-orbital of the metal oxide [5,40].

### 2.3. Mechanistic Approach of Ethylene Adsorption onto Baru-Based Carbon Samples

**Figure 8:**
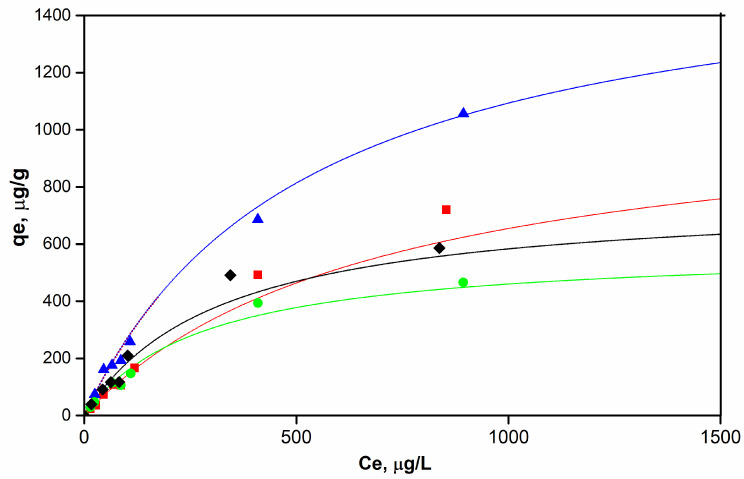
Ethylene adsorption isotherms: (♦) non-activated baru carbon (CB), (▪) baru activated carbon (ACB), (▲) baru activated carbon impregnated with copper oxide (ACB/CuO), (•) activated carbon in oxygen atmosphere (ACB/O_2_). Thin lines are the result of fitting data to Langmuir isotherm model.

Adsorption equilibrium isotherms at 20 °C for the four carbon samples are displayed in Figure 8. Experimental data were fitted to the Langmuir model as global evidence of the surface interactions between ethylene molecules and carbon samples, as follows:

(1) $$q_{e}=\frac{K_{L}\;C_{e}q_{max}}{1+K_{L}\;C_{e}}$$

where q_e_ is the amount of ethylene adsorbed at equilibrium, K_L_ stands for the Langmuir adsorption constant, C_e_ represents the ethylene concentration at equilibrium, and q_max_ is the maximum adsorption capacity. The results depicted in Figure 8 are in agreement with those obtained by other authors for the removal of VOCs using activated carbons modified with metallic oxides, showing forms of a classic type I adsorption isotherm [41].

At constant temperature, the amount of ethylene adsorbed at equilibrium is low when low concentrations are applied. As the applied concentration increases, there is an increase in the adsorption capacities, reaching maximum values for each carbon sample. As can be seen in Table 3, the Langmuir adsorption model provided a good fit to the ethylene adsorption data with R^2^ > 0.98. The ACB/CuO and ACB samples showed the best ethylene adsorption capabilities. A slightly higher performance was observed for the CuO-impregnated sample, due to the new surface sites provided on this carbon sample. Both samples presented the largest volumes of micropores, which indicates that the adsorption performance is also related to the textural properties of the activated carbon. In addition, the CuO-impregnated AC sample resulted in an activated carbon with a smaller average micropore diameter (1.37 nm), which is an important parameter that can explain the interactions between the ethylene molecule (kinetic diameter of approximately 3.9 Å) and the chemical structure of carbon. Micropores that are approximately twice the diameter of ethylene are more accessible, avoiding adsorbate blocking and favoring adsorption performance [42].

**Figure 9:**
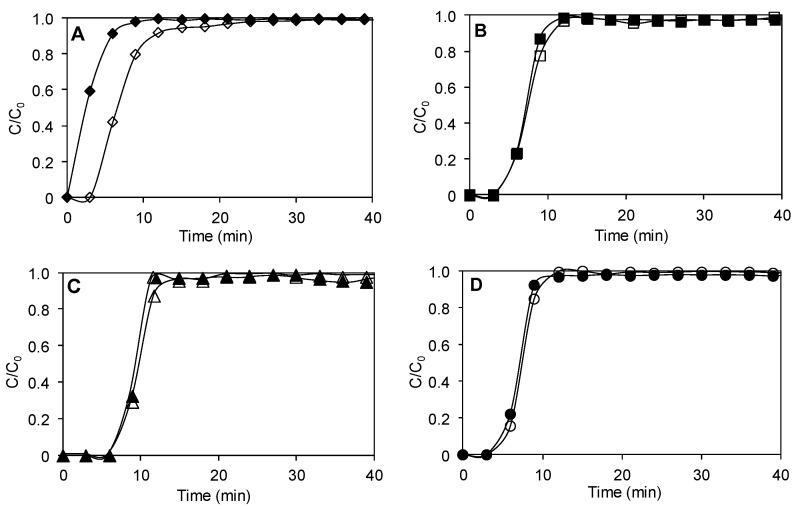
Influence of the presence and absence of moisture on ethylene adsorption: (**A**) non-activated baru carbon, (**B**) baru activated carbon, (**C**) baru activated carbon impregnated with copper oxide, (**D**) activated carbon in oxygen atmosphere.. Experimental conditions: 0.1 g of sample, 25 cm^3^ min^−1^ ethylene (75 μgL^−1^) at 20 °C. ♦ CB, ■ ACB, ▲ ACB/CuO, • ACB/O_2_ (filled markers represent experiments conducted in the presence of moisture (RH 98%) and open markers represent in the absence of moisture).

Furthermore, the results for the influence of the presence of moisture on ethylene adsorption are illustrated in Figure 9. As can be noted, the presence of moisture does not affect ethylene adsorption on all activated carbon samples assessed here. However, a great difference is observed in the case non-activated baru carbon, where a reduction in the adsorption capacity was obtained. These results imply that the surface sites of baru activated carbons where ethylene adsorption occurs are not blocked by the presence of water molecules. Humidity is an important component in fruit storage systems, and below the ideal range (usually ~85%), dehydration and wilting increase [8]. Therefore, adsorbents must be able to adsorb ethylene in high-relative-humidity environments. Activated carbons generated from baru waste appear as alternative adsorbent materials to remove ethylene from closed environments with a high content of humidity.

The results obtained here are in agreement with those obtained by *operando* IR spectroscopy. The removal of ethylene using baru-based activated carbons seems to take place through a combination of adsorption mechanisms that include interactions of ethylene with hydroxyl groups and with copper incorporated on the AC surface. Thus, hydrogen-bonded adducts could be formed between the hydroxyl functional groups on the AC surface and ethylene molecules. Additionally, the π molecular orbital of the adsorbed ethylene donates electron density to the empty s-orbital of the metal oxide, leading to the observed increase in the adsorption capacity of the CuO-impregnated AC sample.

## 3. Materials and Methods

### 3.1. Raw Materials and Reagents

The baru waste was obtained from a local commercial producer located in the state of Minas Gerais, Brazil (17°11′39″ S 44°48′49″ O), in June 2022. Argon (99.9% purity) was supplied by Praxair (Santiago, Chile). Ethylene (C_2_H_4_; 99.99% purity) was provided by Air Liquide S.A. (Houston, TX, USA). Copper (II) nitrate trihydrate (Cu(NO_3_)_2_·3 H_2_O; 99.5%) and phosphoric acid (H_3_PO_4_; 85%) were obtained from Sigma Aldrich, St. Louis, MO, USA.

### 3.2. Preparation of Porous Materials

#### 3.2.1. Preparation and Characterization of the Precursor Material

First, the baru waste was washed with distilled water to remove impurities and then dried in a forced convection oven (TE-394/3, Tecnal, Piracicaba, Brazil) for 48 h at 65 °C. The dried waste was then crushed using a shredder and a chipper (TL 1200, Lippel, Agrolândia, Brazil), and the particle size was reduced to 2 mm. The crushed materials were kept in a desiccator as a raw material precursor to produce activated carbons. The baru waste was characterized for ash content, according to standardized methods [43]. The fractions of the raw lignocellulosic components of the biomass (hemicellulose, cellulose, and lignin) were determined using a protocol established for raw materials by the *Unité d’Amelioration Génétique et Physiologie Forestières* (AGPF) using the *INRA GénoBois* technical platform [18,44]. Thermogravimetric analyses were performed using a DTG thermobalance (60/60H-Shimadzu, Kyoto, Japan), according to a methodology proposed by other authors [18]. The procedure included a temperature rise at a rate of 20 °C min^−1^, from room temperature up to 800 °C (to study a larger temperature domain), followed by a 1 h interval at this final temperature and then cooling to room temperature at 10 °C min^−1^.

#### 3.2.2. Generation of Activated Carbon Materials

Activated carbons were prepared from the precursor material according to a methodology described by other authors [38]. First, the sample was impregnated with phosphoric acid (H_3_PO_4_, 85%) at an impregnation ratio of 1:2 (raw material/acid). Then, the mixture was heated up to 80 °C and kept under mechanical stirring at this temperature for 30 min. The resulting material was filtered and dried in an oven at 110 °C for 15 h. After this step, the impregnated material was carbonized in a tubular oven (FT 1200, Sanchis, Porto Alegre, Brazil) under the following conditions: temperature of 800 °C for 40 min; heating rate of 20 °C min^−1^ under nitrogen flow or in air (160 cm^3^ min^−1^). After that, the carbonized sample was first washed with 37% hydrochloric acid and then with distilled water until the pH was close to neutral. Finally, the activated carbon generated from baru waste was oven-dried at 50 °C until the complete evaporation of water was ensured. Samples activated in nitrogen flow were called ACB, and samples activated in air were named ACB/O_2_. In addition to these samples, non-activated baru carbon (CB) was also obtained. In case of CB, the process is the same as for the preparation of ACB but in the absence of H_3_PO_4_ as activating agent.

### 3.3. Preparation of Baru Activated Carbon Impregnated with Copper Oxide (ACB/CuO)

Surface modification of activated carbon with copper oxide (CuO) was performed using a simple wet impregnation method described elsewhere [14]. In a typical modification procedure, 5.0 g of carbon (ACB) was added to 100 cm^3^ of 0.1 M copper nitrate (Cu(NO_3_)_2_) solution. The mixture was kept under stirring at 25 °C overnight and then filtered and dried at 110 °C for 12 h. Then, the precursor was transferred to a tubular oven and heated from room temperature up to 280 °C at 10 °C min^−1^ under N_2_ flow (200 cm^3^ min^−1^) for 2 h. After cooling, the ACB/CuO sample was obtained.

### 3.4. Characterization of AC Samples

Nitrogen adsorption and desorption isotherms of AC samples were obtained in a Micrometrics ASAP instrument (Gemini V2380, Norcross, GA, USA) at −196 °C. Specific surface area (A_BET_) was determined from the adsorption curve in the range 0.05 ≤ p/p_0_ ≤ 0.15, using the Brunauer–Emmett–Teller (BET) theory. Total pore volume (V_T_) was recorded at p/p_0_ = 0.95, whereas micropore volume (V_micro_) was determined according to Barrett–Joyner–Halenda (BJH) approach [45]. Mesopore volume (V_meso_) was calculated by the difference between V_T_ and V_micro_. Morphology and chemical composition of carbons were determined by field-emission scanning electron microscopy (FESEM) coupled to X-ray energy-dispersive spectroscopy (EDS)—ZEISS Gemini SEM 360 (Jena, Germany). Thermogravimetric analyses (TGA) of the samples were performed on a NET-ZSCH thermobalance ST409PC (Pomerode, Brazil). Samples of 0.025 g of each AC were heated up to 1000 °C (heating rate of 10 °C min^−1^) under N_2_ flow (160 cm^3^ min^−1^), and the change in sample weight in relation to change in temperature was registered (TG curve). A derivative weight loss curve was also obtained as function of temperature (DTG curve). X-ray diffraction (XRD) patterns were obtained on a Bruker D8 Discover diffractometer (Billerica, MA, USA), using a monochromatic radiation from a tube with a copper anode coupled to a Johansson monochromator operating at 40 kV and 40 mA, Bragg Brentano θ–2θ configuration, Lynxeye one-dimensional detector, range of 2θ from 2° to 80°, with a step of 0.01°. The pH of the point of zero charge, defined as the pH at which the carbon surface has a neutral charge, was determined following the procedure described by Kuśmierek et al. [46]. Functional surface groups were directly evaluated by IR spectroscopy (PERKIN ELMER Spectrum 400 FT-IR spectrometer, Waltham, MA, USA) using attenuated total reflectance (ATR) technique in the infrared region of 4000–500 cm^−1^, with a resolution of 4 cm^−1^.

### 3.5. Chemical Surface Interaction Assessments by Operando Transmission IR Spectroscopy

Chemical interactions between the surface groups of ACs and ethylene were monitored as a function of time in a homemade transmittance cell developed by the Catalysis and Spectrochemistry Laboratory (LCS, Caen, France) and set in a Nicolet™ iS™50 spectrometer (Thermo Fisher Scientific Inc., Waltham, MA, USA) equipped with a DTGS detector. Pellets (Ø = 16 mm, m ≈ 20 mg cm^−2^) with a mass of approximately 40 mg (5% *w*/*w* activated carbon/KBr) were formed. A pellet made of KBr was used as a background. Before the measurements, samples were thermally activated inside the cell at 100 °C under Ar flow (5 cm^3^ min^−1^) for 15 min. Then, a spectrum of the adsorbate free sample was measured. After that, a stream made of 1% ethylene in Ar was passed through the pellet for 200 min. Subsequently, the diluted ethylene stream was changed to pure ethylene, and the analyses continued until reaching saturation. IR transmittance spectra were registered as a function of time. Spectra were obtained with 60 scans at a resolution of 4 cm^−1^ in a range from 4000 to 500 cm^−1^.

### 3.6. Determination of Ethylene Adsorption Isotherms

Ethylene adsorption isotherms were conducted through dynamic adsorption tests on a quartz fixed-bed flow adsorber, as per the procedure described by Abreu et al. [5]. The adsorber was loaded with 0.1 g of sample. Before contact with ethylene, the samples were thermally degassed at 150 °C (3 °C min^−1^) under argon flow (100 cm^3^ min^−1^) for 1 h. The inlet concentration of ethylene was fixed by diluting a pure stream of C_2_H_4_ with argon using mass flow controllers. A total flow rate of 25 cm^3^ min^−1^ of ethylene at the desired concentration was continuously delivered over the AC sample. The ethylene concentration was determined by gas chromatography (PERKIN ELMER CLARUS 500 gas chromatograph, Waltham, MA, USA) equipped with a flame ionization detector (FID). The adsorption capacity of each AC sample toward ethylene was determined by calculating the areas of different breakthrough curves. For experiments in the presence of humidity, the entire stream was bubbled in a humidification chamber maintained with water at 293 K, allowing for a constant wet flow rate at 98% relative humidity (RH). The adsorption capacities of AC samples towards ethylene (q_ethylene_, μmol_ethylene_ g carbon^−1^) were determined by calculating the areas of different breakthrough curves, as follows:

(2) $$q_{ethylene}=\frac{F\;C_{in}}{m}\int_{0}^{t_{s}}\left(1-\frac{C_{out_{t}}}{C_{in}}\right)dt$$

where F (cm^3^ min^−1^) is the gas flow rate, m (g) is the mass of AC placed inside the quartz adsorber, C_in_ and C_out_ (μmol dm^−3^) are the ethylene inlet and outlet concentrations as a function of time, respectively, and t_s_ (min) is the adsorption time to reach saturation.

## 4. Conclusions

Baru waste has the potential to be used as a low-cost precursor in the generation of activated carbons, since it has significant levels of lignin, cellulose, and hemicellulose and a low ash content, which leads to a considerable carbonization yield, even in high-temperature conditions. It was possible to produce activated carbons and carry out surface modifications by impregnation with copper species, which were confirmed by characterization analyses (XRD and FESEM/EDS). Surface modification with copper oxide improved the ethylene adsorption capacity compared to other generated porous materials. The efficient adsorption of ethylene on the CuO-impregnated AC sample was favored by the surface acidity of this AC, where hydrogen-bonded adducts were formed due to the interactions of the surface OH groups and ethylene molecules and also by the interactions between the empty s-orbital of the copper oxide and π-electrons of the C=C bonds of the ethylene molecules. The presence of moisture did not affect the adsorption capacity of baru activated carbon samples, which makes this type of activated carbon an excellent adsorbent option to eliminate ethylene from closed containers with a high content of humidity. Future works should focus on evaluating the regeneration performance and operating conditions for the practical application of this new adsorbent during the storage of climacteric fruit.

## Figures and Tables

**Table 1:** Textural characteristics of adsorbent materials.

Samples	S_BET_ [m^2^ g^−1^]	V_T_ [cm^3^ g^−1^]	V_meso_ [cm^3^ g^−1^]	V_micro_ [cm^3^ g^−1^]	L_0_ [nm]	pH_PZC_
CB	291	0.16	0.05	0.11	4.35	6.98
ACB	886	0.46	0.09	0.37	1.72	3.81
ACB/CuO	628	0.34	0.04	0.30	1.37	3.65
ACB/O_2_	747	0.39	0.21	0.18	2.71	3.53

S_BET_ = BET surface área; V_T_ = total pore volume; V_micro_ = micropore volume; V_meso_ = mesopore volume; L_0_ = average micropore width.

**Table 2:** Comparison of S_BET_ surface areas of different copper-modified porous materials reported in the literature.

Precursor	Adsorbents	S_BET_ [m^2^ g^−1^]	References
Baru activated carbon	ACB/CuO	628	This work
Natural zeolite	NH_4_Z_2_-Cu	351	[5]
Commercial activated carbon	10-CuO/AC-800	667	[14]
Commercial activated carbon	CuO/AC08	947	[13]
Porous boron nitride	BN-Cu	626	[34]

**Table 3:** Langmuir parameters for ethylene adsorption at 20 °C on non-activated baru carbon and modified carbon samples.

Samples	q_max_ [μg g^−1^]	K_L_ [dm^3^ μg^−1^]	R^2^
CB	769	0.00314	0.98
ACB	1111	0.00143	0.98
ACB/CuO	1667	0.00191	0.98
ACB/O_2_	588	0.00358	0.99

## Data Availability

The data that support the findings of this study are available from the corresponding author upon reasonable request.
